# Fibrosis in fat: From other diseases to Crohn’s disease

**DOI:** 10.3389/fimmu.2022.935275

**Published:** 2022-08-25

**Authors:** Shanshan Xiong, Jinyu Tan, Yu Wang, Jinshen He, Fan Hu, Xiaomin Wu, Zishan Liu, Sinan Lin, Xuehua Li, Zhihui Chen, Ren Mao

**Affiliations:** ^1^ Department of Gastroenterology, The First Affiliated Hospital of Sun Yat-sen University, Guangzhou, China; ^2^ Department of Radiology, The First Affiliated Hospital of Sun Yat-sen University, Guangzhou, China; ^3^ Gastrointestinal Surgery Center, The First Affiliated Hospital of Sun Yat-sen University, Guangzhou, China; ^4^ Department of Gastroenterology, Huidong People’s Hospital, Huizhou, China

**Keywords:** creeping fat, adipose tissue, fibrosis, extracellular matrix, Crohn’s disease

## Abstract

Creeping fat is a specific feature of Crohn’s disease (CD) and is characterized by mesenteric fat wrapping around the intestine. It highly correlates with intestinal transmural inflammation, muscular hypertrophy, fibrosis, and stricture formation. However, the pathogenesis of creeping fat remains unclear. Molecular crosstalk exists between mesenteric fat and the intestine. Indeed, creeping fat contains different types of cells, including adipocytes and immune cells. These cell types can produce various cytokines, fatty acids, and growth factors, which affect the mesenteric fat function and modulate intestinal inflammation and immunity. Moreover, adipocyte progenitors can produce extracellular matrix to adapt to fat expansion. Previous studies have shown that fat fibrosis is an important feature of adipose tissue malfunction and exists in other diseases, including metabolic disorders, cancer, atrial fibrillation, and osteoarthritis. Furthermore, histological sections of CD showed fibrosis in the creeping fat. However, the role of fibrosis in the mesenteric fat of CD is not well understood. In this review, we summarized the possible mechanisms of fat fibrosis and its impact on other diseases. More specifically, we illustrated the role of various cells (adipocyte progenitors, macrophages, mast cells, and group 1 innate lymphoid cells) and molecules (including hypoxia-inducible factor 1-alpha, transforming growth factor-beta, platelet-derived growth factor, and peroxisome proliferator-activated receptor-gamma) in the pathogenesis of fat fibrosis in other diseases to understand the role of creeping fat fibrosis in CD pathogenesis. Future research will provide key information to decipher the role of fat fibrosis in creeping fat formation and intestinal damage, thereby helping us identify novel targets for the diagnosis and treatment of CD.

## 1 Introduction

Crohn’s disease (CD) is a chronic inflammatory disease that can affect any part of the digestive tract, and its pathophysiological mechanism is complex (1). Within 10 years of its diagnosis, approximately 50% of the patients need surgical treatment. Moreover, the disease cannot be cured with a high disability rate, placing a heavy burden on patients, families, and society (2). In severe cases of CD, the mesenteric white adipose tissue surrounding the diseased intestinal wall extends from the mesenteric attachment and partially covers the intestinal circumference to form “creeping fat” (3, 4). Creeping fat is a unique pathological feature of CD, and its presence correlates with intestinal transmural inflammation, muscular hypertrophy, fibrosis, and stricture formation (5–7). Moreover, this structure helps recognize the site of the most severe lesions, fibrosis, and stenosis during surgery, and resection of the diseased mesentery reduces the postoperative recurrence of CD (8). However, the pathogenesis of creeping fats remains unclear.

**Figure 1:**
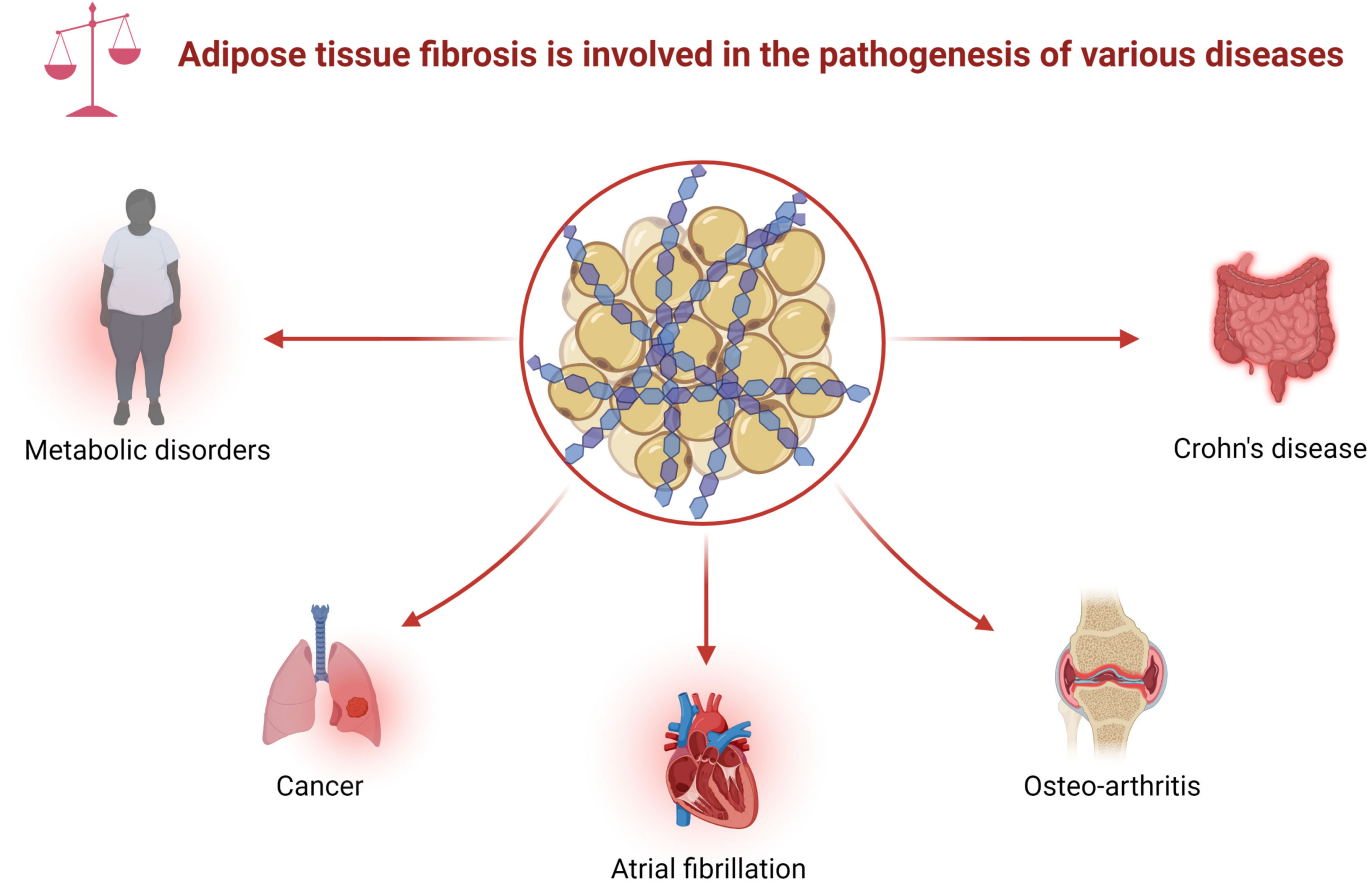
Adipose tissue fibrosis is involved in the pathogenesis of various diseases. Adipose tissue fibrosis, defined as excessive deposition of ECM in adipose tissue, appears in various diseases, including metabolic disorders, cancer, atrial fibrillation, osteoarthritis, and Crohn’s disease. ECM, extracellular matrix.

With the rise in metabolic diseases, researchers are focusing on the function of adipose tissue, including creeping fat. There is molecular crosstalk between the mesenteric fat and the intestinal wall (9–11). Creeping fat contains diverse cell types, including adipocyte progenitors, adipocytes, and immune cells. These cells produce various cytokines, fatty acids, or growth factors, which affect mesenteric fat function, which, in turn, can modulate intestinal inflammation and immunity (12). For instance, some adipokines, including adiponectin and leptin, shape the local macrophages to mostly the M2 subtype, suggesting a protective role of mesenteric fat in CD (10). The free fatty acids secreted by the adipocytes in creeping fat modulate intestinal smooth muscle proliferation, which further promotes stricture formation (6). Moreover, adipocyte progenitors can produce extracellular matrix (ECM) to adapt to fat expansion (13). Abnormal ECM deposition, a feature of fibrosis development in adipose tissue, is associated with tissue inflammation and adipose tissue malfunction in metabolic diseases (14, 15). Indeed, fat fibrosis exists in various diseases, such as metabolic disorders, tumors, atrial fibrillation (AF), and immune diseases (**Figure 1**) (14). Furthermore, histological sections of CD showed fibrosis in the creeping fat (16, 17). However, the role of fat fibrosis in the pathogenesis of creeping fat has gained little attention due to the lack of an ideal preclinical model.

In this review, we aimed to discuss the possible mechanisms of adipose tissue fibrosis and its impact on other diseases. We believe that the mechanism from these disorders may be pivotal to elucidate the role of adipose tissue fibrosis in the pathogenesis of CD. It will help researchers get a comprehensive overview of the topic and provide directions for conducting future research to identify novel targets for CD diagnosis and treatment.

## 2 Definition and ECM characteristics of adipose tissue fibrosis

### 2.1 Definition

Adipose tissue fibrosis is the excessive deposition of ECM in adipose tissue. Although the ECM composition of adipose tissue is similar to that of other tissues, the relative content of ECM proteins may differ (18). In addition, there are significant differences in the proportion of ECM proteins between the adipose tissues of lean and obese individuals (19). The size and morphology of adipocytes also change in expanded fat, and there is no clear understanding of these specific manifestations.

### 2.2 ECM characteristics

The most abundant ECM proteins in adipose tissue are members of the collagen family, including collagen I, III, and V, and microfibrillar collagen IV (20). Type I collagen is the predominant one that maintains the structure and function of adipose tissue, along with other fibril-forming molecules (21). Collagen IV is the main component of the basement membrane and is necessary for adipocyte survival. It provides cellular structural support and interacts with integrins to transfer signals (22, 23). Increased collagen VI levels are observed in the fibrotic adipose tissue of both rodents and humans during metabolic challenges (24). Compared with other ECM proteins, collagen VI is more specific to adipose tissue and is critical for adipose tissue fibrosis and dysfunction (24). Collagen VI consists of three subunits, α1(VI), α2(VI), and α3(VI), which are highly regulated from the gene to the post-translational levels (25). Morphologically, collagen VI-null mice in the high-fat diet and ob/ob mutation group have an increased adipocyte cell size and reduced necrotic cell death and inflammation in adipose tissue (24). In addition, the glucose clearance rate, lipid metabolic parameters, and insulin signaling dramatically improved in collagen VI-null mice (24). These findings indicate that adipose tissue fibrosis caused by type VI collagen is associated with systemic and local metabolic disorders (23).

Moreover, endotrophin, a post-transcriptional protein-derived product of collagen VI, is overexpressed in the adipose tissue of ob/ob mice (26). Endotrophin plays a crucial role in tumor development (27, 28) and adipose tissue fibrosis and inflammation (26). Mechanistically, collagen VI and endotrophin cooperatively regulate the adipogenic and lipolytic capacity of adipocytes *via* the MAPK signaling pathway, regardless of their role in structural support in obesity-related metabolic diseases (29).

### 2.3 Regulation of collagen synthesis and degradation in adipose tissue

Collagen quantity is determined by the balance of enzymes that promote ECM synthesis and degradation. The synthesis enzymes include intracellular enzymes that participate in the processing of ECM protein precursors and extracellular inhibitors of degrading enzymes (18). Degradation enzymes include the fibrinolytic system, matrix metalloproteinase (MMP), and tissue inhibitors of MMPs (TIMPs) (18, 30). In this review, we will focus on the MMP system since its level significantly alters in the adipose tissue during adipose tissue expansion, and it also plays a role in cleaving collagen, thereby remodeling the ECM (31). Moreover, the MMP system causes adipose dysfunction and inflammation during tumor growth regulation (32).

In nutritionally induced obese mice, the mRNA expression of *MMP-3, MMP-11, MMP-12, MMP-13, MMP-14*, and *TIMP-1* is upregulated, while that of *MMP-7*, *MMP-9, MMP-16, MMP-24*, and *TIMP-4* is downregulated (32). *MMP-2* and *MMP-9* levels are also significantly higher in obese patients than in the controls (33–35). MMP-2 inhibitors prevent 3T3-L1 preadipocyte differentiation in a dose-dependent manner, and increased expression of MMP-2 and MMP-9 is associated with the loss of basement membrane type IV collagen (36, 37). Circulating MMP-9 also increases in obese patients and is associated with insulin resistance (38). In addition, membrane type 1 MMP (MT1-MMP, also known as MMP14) is critical in adipose tissue ECM remodeling. MMP14 activates MMP-2 and forms ternary complexes important for basement membrane remodeling during adipogenesis with TIMP-2, MMP-2, or MMP-9 (39, 40). It can also regulate the cleavage of collagen I. Furthermore, the MMP-14-null mice develop soft tissue fibrosis, which may be related to collagen renewal disorders (41). Simply put, MMP14 is crucial for adipocyte differentiation and collagen synthesis, and its absence affects the function of adipose tissue.

## 3 Cellular and molecular mechanisms of adipose tissue fibrosis

### 3.1 Cells

Myofibroblasts are pivotal for ECM production and remodeling (42). However, little is known about its role in adipose tissue fibrosis. Adipocyte progenitors can differentiate into myofibroblasts, which drive ECM synthesis in obesity (14). Platelet-derived growth factor receptor alpha (PDGFRα)+ adipocyte progenitors can obtain myofibroblast phenotypes and produce the highest levels of fibrosis markers in fibrotic adipose tissue (43). In addition, a subgroup of PDGFRα+ cells with high CD9 expression is associated with white adipose tissue fibrosis and metabolic disorders (43).

The adipose tissue of obese individuals has a significantly high number of macrophages than in control, and these macrophages are associated with inflammation and insulin resistance (44). There are two main types of macrophages: M1 and M2 (45). M1 macrophages promote inflammation, leading to insulin resistance in the adipose tissue of obese individuals (46). However, M2 macrophages can inhibit M1 macrophages, which maintain adipose tissue homeostasis (47). Furthermore, a crown-like structure represents macrophages clustering in dead and dying adipocytes (48). Studies have shown that macrophages in the crown-like structure are mainly M1, while M2 macrophages are abundant in adipocytes in fibrotic areas (49). These differences in the distribution of macrophages may be associated with differences in their functions. The co-culture of M1 macrophages with adipocytes leads to a more M2 phenotype, suggesting that inflammation and fibrosis coexist in adipose tissues (49). Interestingly, macrophage-inducible C-type lectin (Mincle) modulates macrophage function and correlates with myofibroblast activation and ECM remodeling, and mincle-knock out mice are protected against adipose tissue fibrosis (50, 51). Moreover, infiltrating macrophages in adipose tissue can release signals that attract fibroblasts and regulate adipose tissue fibrosis (14).

Apart from macrophages, mast cells are also present in adipose tissue and are associated with collagen accumulation and adipose tissue remodeling. Immature progenitor cells are released from the bone marrow and settle in vascularized tissue to mature within the blood (52, 53). The abundance of mast cells appears to increase in both animal models and patients with obesity, implicating their potential role in metabolic diseases (54–56). Furthermore, mast cells that infiltrate obese adipose tissue secrete mast cell protease 6 and induce collagen V expression, contributing to adipose tissue fibrosis and accelerating insulin resistance by inhibiting preadipocyte differentiation (57).

Recently, group 1 innate lymphoid cells (ILC1s) have been shown to be involved in the pathogenesis of adipose tissue fibrosis. Wang et al. showed that the number of ILC1 in adipose tissue increases in obese patients with type 2 diabetes and induces fat fibrosis in an interferon-γ-dependent manner (58). In addition, the reconstitution of adipose ILC1s by adoptive transfer in Prkdc-/- IL2rg-/- mice (immunodeficient mice) promotes adipose tissue fibrosis *via* transforming growth factor-beta 1 (TGF-β1) signaling, whereas inhibiting the accumulation of ILC1s can reduce fibrosis in adipose tissue and improve glucose tolerance (58).

In general, adipose tissue fibrosis is associated with the differentiation of adipocyte progenitors and the infiltration of various immune cells (14). Poorly differentiated mesenteric adipocytes and enriched immune cells are also confirmed in the mesenteric adipose tissue of CD patients (59, 60). Furthermore, our group previously showed that intestinal muscle cells and preadipocytes in CD patients interacted with each other and activated muscle cells could produce an ECM scaffold (6). Recently, another team highlighted the role of TLR4-mediated macrophages in mesenteric adipocyte dysfunction (61). All in all, creeping fat fibrosis is a complex process involving various cells, including fibroblasts, muscle cells, preadipocytes, and macrophages. The specific cellular mechanism of fat fibrosis still needs further investigation.

### 3.2 Related signal molecules

#### 3.2.1 Hypoxia-inducible factor 1-alpha

Adipose tissue expansion leads to a hypoxic status because angiogenesis fails to catch up with tissue growth. One of the key mediators of this process is hypoxia-inducible factor 1-alpha (HIF1α) (62). HIF1α cannot stimulate the expected angiogenesis program but can induce adipose tissue fibrosis and insulin resistance (63). Its transcriptional target lysyl oxidase is significantly increased in leptin-deficient ob/ob mice and crosslinks collagen I and III to form fibrillar collagen (63). Subsequently, the M1 macrophages are recruited, which release various inflammatory mediators, such as interleukin (IL)-6, monocyte chemoattractant protein 1, tumor necrosis factor-alpha, and IL-1β, and induce inflammation (64). Furthermore, a selective HIF1α inhibitor (PX-478) or a negative HIF1α mutation could suppress the formation of adipose tissue fibrosis in high-fat diet-fed mice and improve the metabolic state (65). Consistent with the findings above, Zuo et al. found that mesenteric adipose tissue contiguous with the involved intestine has a higher level of HIF1α than that in the uninvolved intestine (59). Although the exact mechanisms of the changes observed are still not clear, previous literatures on HIF1α give clues to the study of adipose tissue fibrosis in CD.

#### 3.2.2 TGF-β

The TGF-β superfamily proteins are crucial regulators of adipose tissue remodeling. TGF-β1 and activin A belong to this superfamily and assist human adipose progenitors to acquire the myofibroblast phenotype and prevent their differentiation into adipocytes (66, 67). Likewise, when exposed to TGF-β1, murine-derived 3T3‐L1 preadipocytes synthesize more ECM proteins and reduce differentiation (68). The response to TGF-β is mediated by SMAD2, SMAD3, and SMAD4, which subsequently activate the fibrotic genes, such as collagen, fibronectin, and ECM remodeling enzymes (69). M2 macrophages in adipose tissue express high levels of TGF-β, which can be enhanced by co-culture with adipocytes. The expression of downstream effectors, such as phosphorylated SMAD, plasminogen activator inhibitor-1, and collagens, is also increased in macrophages and adipocytes (49, 70). Metformin can decrease ECM deposition in adipose tissue by activating AMPK signaling and inhibiting TGF-β1/Smad3 signaling, which improves fibrosis and prevents uncontrolled adipose tissue expansion in metabolic disorders (71). As for CD, studies have shown that the level of TGF-β in the mesenteric adipose tissue was significantly increased, with the Smad2/3 signaling pathway activated. This change also led to excessive ECM synthesis in the mesenteric adipose tissue (59, 61).

#### 3.2.3 Platelet-derived growth factor

PDGF is another key fibrosis signaling molecule. It combines two conserved tyrosine kinase receptors: PDGFRα and PDGFRβ, which play important roles in the proliferative profibrotic phenotype (72). Adipocyte progenitors express both PDGFRα and PDGFRβ.

When PDGFRα signaling is activated, adipocyte progenitors synthesize ECM and function like profibrotic cells, contributing to pathological remodeling and adipose tissue dysfunction in obesity (43). PDGFα signaling typically correlates with Zfp521 overexpression (73). However, how PDGFα signal transduction converts progenitors into a fibrotic phenotype is yet to be completely elucidated. A study suggested that PDGFα may act by upregulating the mTOR mRNA translation and ribosomal biogenesis signaling pathways and control the expression of the imprinted gene network related to cell growth and tissue homeostasis (74).

Furthermore, PDGFRβ inhibits the adipogenic potential of progenitors and promotes liver and kidney fibrosis (75). However, there is no evidence of its direct role in driving adipose tissue fibrosis (75). A study showed that adiponectin-positive intradermal adipose tissue can be transformed into myofibroblasts in bleomycin-treated mice (76). Hence, it can be speculated that the reactivation of these receptors under pathological conditions is related to fibrosis or tissue dysfunction.

According to the current available literature, no relevant research exists to study the role of PDGF in CD fat fibrosis. Since PDGF has shown its exact pro-fibrotic role in other organs, future study is needed to elucidate its mechanism in mesenteric adipose tissue fibrosis of CD.

#### 3.2.4 Peroxisome proliferator-activated receptor-gamma

The peroxisome proliferator-activated receptor (PPAR) family comprises three members: PPAR-α, PPAR-δ, and PPAR-γ. Among them, PPAR-γ is mainly present in adipose tissue and regulates adipogenesis and lipid metabolism (77). When treated with a PPARγ agonist, diabetic db/db mice showed lower collagen expression, suggesting an anti-fibrotic capacity of PPAR**γ** (63). Furthermore, a study revealed an association between PPARγ2 and HIF1α, since HIF1α attenuates adipogenesis and promotes white adipose tissue fibrosis in obesity by driving PPARγ S112 phosphorylation *via* autocrine/paracrine signaling. The blocking effect can be imitated by an antagonist of PDGFR (imatinib). Therefore, PDGFR signaling may play a key role in HIF1α activation and inhibition of PPARγ activity (78). Although there is no statistical significance, tissue concentrations of PPARγ were also increased in creeping fat adjacent to the lesion (61). It is reasonable to speculate that PPARγ may also be involved in the fibrotic process of mesenteric adipose tissue in CD patients.

#### 3.2.5 Other signaling molecules

**Figure 2:**
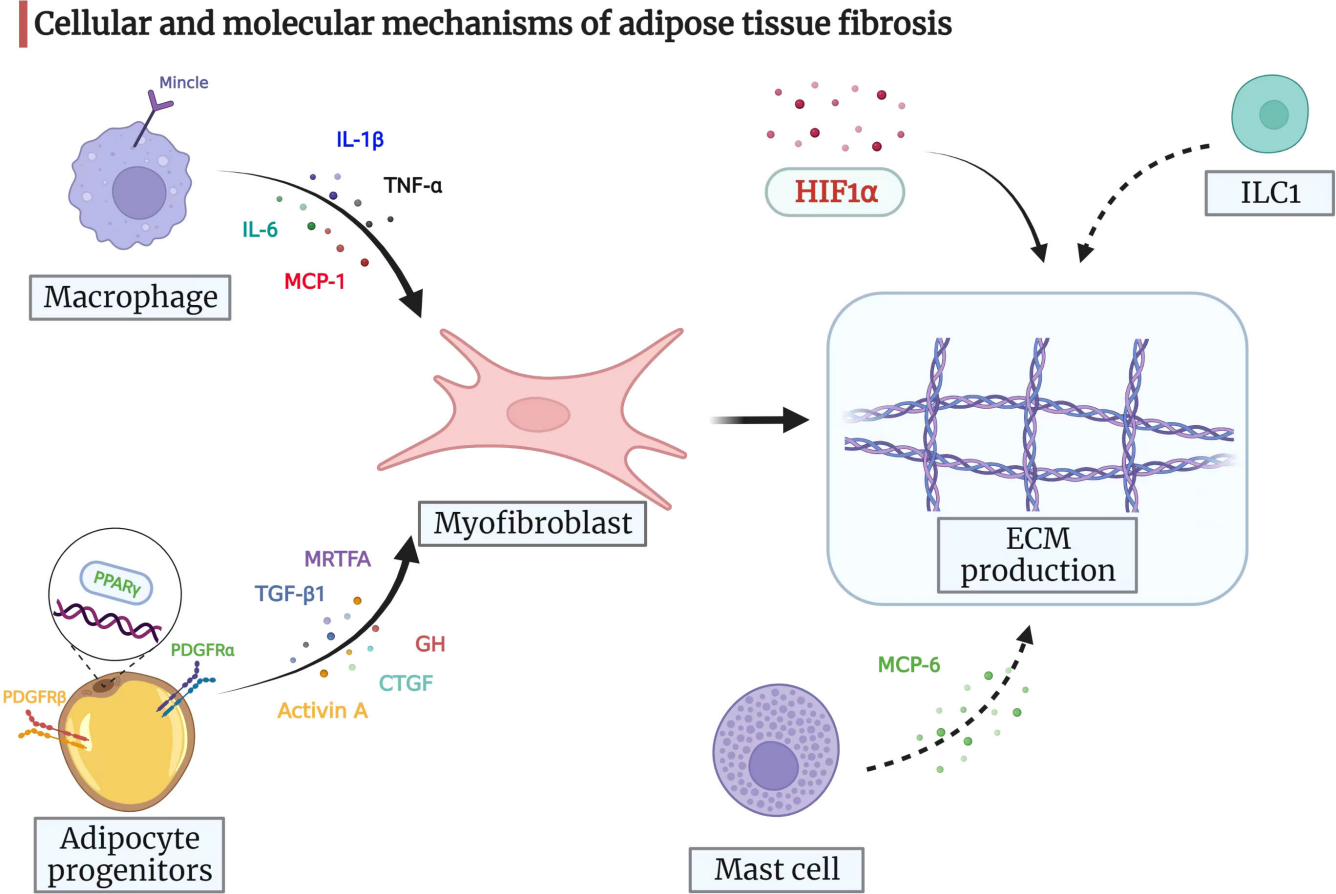
Cellular and molecular mechanisms of adipose tissue fibrosis. Myofibroblasts are pivotal for ECM production and remodeling. Adipocyte progenitors can differentiate into myofibroblasts, which then drive ECM synthesis. Adipocyte progenitors express both PDGFRα and PDGFRβ. When PDGFRα signaling is activated, adipocyte progenitors synthesize ECM and function as profibrotic cells. PDGFRβ inhibits the adipogenic potential of progenitors. The nuclear receptor PPARγ also regulates adipogenesis with anti-fibrotic potential. Moreover, TGF-β1, activin A, CTGF, GH, and MRTFA may drive adipose progenitors to acquire a myofibroblast phenotype and prevent differentiation into adipocytes under certain circumstances. The infiltrating macrophages in adipose tissue can release signals, such as IL-6, MCP-1, TNF-α, and IL-1β, which attract fibroblasts and regulate adipose tissue fibrosis. In addition, macrophage-inducible C-type lectin (Mincle) modulates macrophage function and correlates with myofibroblast activation and ECM remodeling. Mast cells secrete MCP-6 and induce collagen V expression, contributing to adipose tissue fibrosis and accelerating insulin resistance by inhibiting preadipocyte differentiation. ILC1 in adipose tissue induces fat fibrosis in an IFN-γ-dependent manner. HIF1α promotes adipose tissue fibrosis and is a potential therapeutic target for adipose tissue fibrosis and associated metabolic disorders. ECM, extracellular matrix; PDGF, platelet-derived growth factor; PPARγ, peroxisome proliferator-activated receptor-gamma; TGF-β1, transforming growth factor-beta 1; CTGF, connective tissue growth factor; GH, growth hormone; MRTFA, myocardin-related transcription factor A; IL-6, interleukin-6; MCP-1, monocyte chemoattractant protein-1; TNF-α, tumor necrosis factor-alpha; IL-1β: interleukin-1beta; MCP-6, mast cell protease 6; ILC1s, group 1 innate lymphoid cells; IFN-γ, interferon-gamma; HIF1α, hypoxia-inducible factor 1-alpha.

There are multiple other signaling molecules involved in the process of adipose tissue fibrosis, such as connective tissue growth factor (79, 80), growth hormone (81), and myocardin-related transcription factor A (82). However, no reliable conclusions could be drawn based on the results of previous studies. Nevertheless, the findings from the above-mentioned studies show that adipose tissue fibrosis is driven by an imbalance between the fibrogenic and adipogenic potential of adipose tissue progenitors. The cellular and molecular mechanisms of adipose tissue fibrosis are represented in a pictorial form in **Figure 2**, providing clues to the molecular mechanism of mesenteric adipose tissue fibrosis in CD.

## 4 Implications from other diseases

Adipose tissue fibrosis has been observed in various diseases, including obesity, cancer, and arthritis. Information from these disorders may be pivotal to understanding the role of adipose tissue fibrosis in CD pathogenesis.

### 4.1 Metabolic disorders

Adipose tissue in obese patients exhibits excessive synthesis of fibrotic tissues, associated with phenotypic changes in preadipocyte and pro-inflammatory environments (83). Among obese patients, a subset is considered to be metabolically healthy, while the other subset might be affected by adipose tissue fibrosis (84).

Fibrosis in visceral adipose tissue may have a positive effect on metabolism. In obese patients, the degree of omental fibrosis negatively correlates with the size of the omental fat cells (19). Compared with the adipose tissue in non-diabetic subjects, the degree of omental adipose tissue fibrosis in the adipose tissue, the frequency of preadipocytes, and expression of fibrotic genes are lower and the fat cells are larger in diabetic subjects (85). Another study further supported the link between human omental fat fibrosis and metabolic outcomes. The tensile strength of adipose tissue was used as a proxy for the severity of fibrosis, and the tensile strength of omental adipose tissue decreased in obese subjects with type 2 diabetes than in healthy obese subjects (86). Omental fibrosis also has a more positive effect on lipid metabolism since it negatively correlates with circulating triglyceride levels and positively correlates with high-density lipoprotein cholesterol levels (19). These data indicate that omental fibrosis may be an adaptive phenomenon that can limit the expansion of fat cells and help reduce the negative effects of adipocyte hypertrophy on metabolism.

However, the situation is different for subcutaneous fat. When matched for body mass index, obese patients with insulin resistance show increased expression of ECM markers in subcutaneous fat than in insulin-sensitive obese subjects (87). This result was confirmed in another study on non-diabetic subjects (88). Similar conclusions have been reached by animal studies (24). Furthermore, it has been demonstrated that lacking collagen VI can improve the body’s energy homeostasis (24). This suggests that the increase in the ECM of adipose tissue, especially in subcutaneous fat, is not only caused by obesity but may also be critical for insulin resistance (87).

Therefore, omental and subcutaneous fats exert different effects on metabolism. However, inconsistent conclusions have been drawn from different studies of omental fat (14). Moreover, the influence of fat fibrosis on metabolism is very complicated and most likely related to the location of fat and other possible factors.

### 4.2 Cancer

Obesity is associated with an increased risk of cancer development and a poor prognosis, and adipose tissue fibrosis may be a contributing factor. Both myofibroblasts and fibroblasts are more prevalent in histologically normal breast tissues closer to the edge of breast adenocarcinoma (89). Bo et al. found that obesity increases interstitial fibrosis, which stimulates breast cancer growth *via* altered mechanosignaling (90). This finding illustrated that obesity-associated ECM could drive the tumorigenic potential of premalignant cells (90).

Another group has investigated the interaction between tumor cells, stromal cells, and adipocytes. Collagen VI is abundantly expressed in adipocytes and can promote tumor growth at its early stages (91). Meanwhile, endotrophin, a cleavage product of the COL6α3 chain, enhances fibrosis and is associated with mammary tumor growth. These effects are partially mediated by an enhanced TGF-β signaling (27). Furthermore, Incio et al. showed that obesity-induced desmoplasia led to tumor progression and poor response to chemotherapy in pancreatic ductal adenocarcinoma (92).

Interestingly, fat fibrosis has also been associated with cancer cachexia. Increased collagen and fibers are found in the adipose tissue of cancer cachexia than in weight-stable cancer patients and controls (93). This phenomenon is associated with altered TGF-β signaling, which can affect the structure and function of adipose tissue (93).

### 4.3 Atrial fibrillation

AF is the most common type of arrhythmia. A French group obtained epicardial adipose tissue and thoracic subcutaneous fat samples from 41 patients (94). They showed that the differentially expressed genes were related to ECM remodeling, inflammation, infection, and thrombosis. However, AF was present only in a subset of the study population (94). Furthermore, Abe et al. collected left atrial appendage samples from 59 patients with AF during surgery (95). They showed that fibrotic remodeling of epicardial adipose tissue was associated with left atrial myocardial fibrosis in these patients (95). In another group, Haemers et al. collected atrial samples from 92 patients with AF, analyzed the fibrosis of subepicardial fatty infiltrates, and showed that fibrosis of the fatty infiltrates was predominant in the patients with permanent AF (96) Moreover, cytotoxic lymphocyte and adipocyte cell death may also be involved in this process (96).

### 4.4 Osteoarthritis

Adipose tissue fibrosis is also involved in the pathogenesis of osteoarthritis. Eymard et al. obtained intra-articular and subcutaneous adipose tissues from patients with osteoarthritis during knee or hip replacement and showed that fibrosis, vascularization, and immune cell infiltration were higher in the intra-articular adipose tissue than in the subcutaneous adipose tissue (97). Moreover, the levels of cytokines, such as IL-6, IL-8, and prostaglandin E_2_ also increased (97). Another study focused on a mouse model of early osteoarthritis with 20 weeks of feeding a high-fat diet (98). The high-fat diet did not alter inflammation and macrophage infiltration but increased the infrapatellar fat pad fibrosis, suggesting that the intra-articular adipocyte is a distinct cell type (98). However, the underlying mechanism of how a high-fat diet increased infrapatellar fat pad fibrosis is under investigation.

To sum up, fat fibrosis correlates with the pathogenesis, disease activity, and prognosis of different kinds of diseases. Though it may be different from that in CD, this information provides useful tools, techniques, and direction to decipher the role of fat fibrosis in CD.

## 5 Fat fibrosis in CD itself

Since fat fibrosis is involved in various diseases, it is important to elucidate the occurrence and pathogenesis of fibrosis in the creeping fat of CD. In 2003, Geboes et al. mentioned that “fibrous strands are present in the mesenteric fat, irradiating from the intestine and surrounding thickened, hypertrophied fat lobules” (16). However, this area has received little attention to date since it is challenging to establish an ideal animal model to recapitulate creeping fat fibrosis in humans.

Our team used human CD paired samples to investigate the transcriptional signature of these “fibrous strands” (12). Compared with material from low fibrous band samples, the high fibrous band samples were enriched for mRNAs encoded by 661 genes (*p* < 0.05 and fold change ≥2), including the genes with known roles in fibrosis, such as *COL1A1, FAP, COL6A3, COL1A2, COL5A1*, and *MMP2*. We also created a novel mouse model using repeated colonic biopsies for functional studies (12). In this newly established model, the mucosa was injured, and fat accumulation was detected around the intestine in C57BL/6J mice, mimicking the gross features of creeping fat in CD (12). Histological analysis indicated that fibrosis extended into the mesenteric adipose tissue. Finally, we generated a 24-gene set list (including *COL1A1, COL5A1, LUM, MMP2*, and *FAP*) using both the human CD dataset and the mouse model and linked the list to inflammatory fibroblasts and treatment response (12). Although our study has expanded the knowledge on fibrosis in creeping fat, much remains to be investigated.

Ha et al. reported an altered Schaedler flora in mice with *Clostridium innocuum* and found mesenteric adipose tissue expansion in both dextran sulfate sodium-treated and untreated groups (99). They also successfully isolated bacteria in mesenteric adipose tissue, indicating that *C. innocuum* translocated to mesenteric adipose tissue and promoted adipose tissue expansion (99). Single-cell RNA sequencing showed that both profibrotic and pro-adipogenic signals were present in creeping fat (99). Therefore, bacterial translocation may play a role in the formation of creeping fat (99). Moreover, another study showed that mesenteric microbiota from CD patients promotes intestinal inflammation in mice (100).

**Figure 3:**
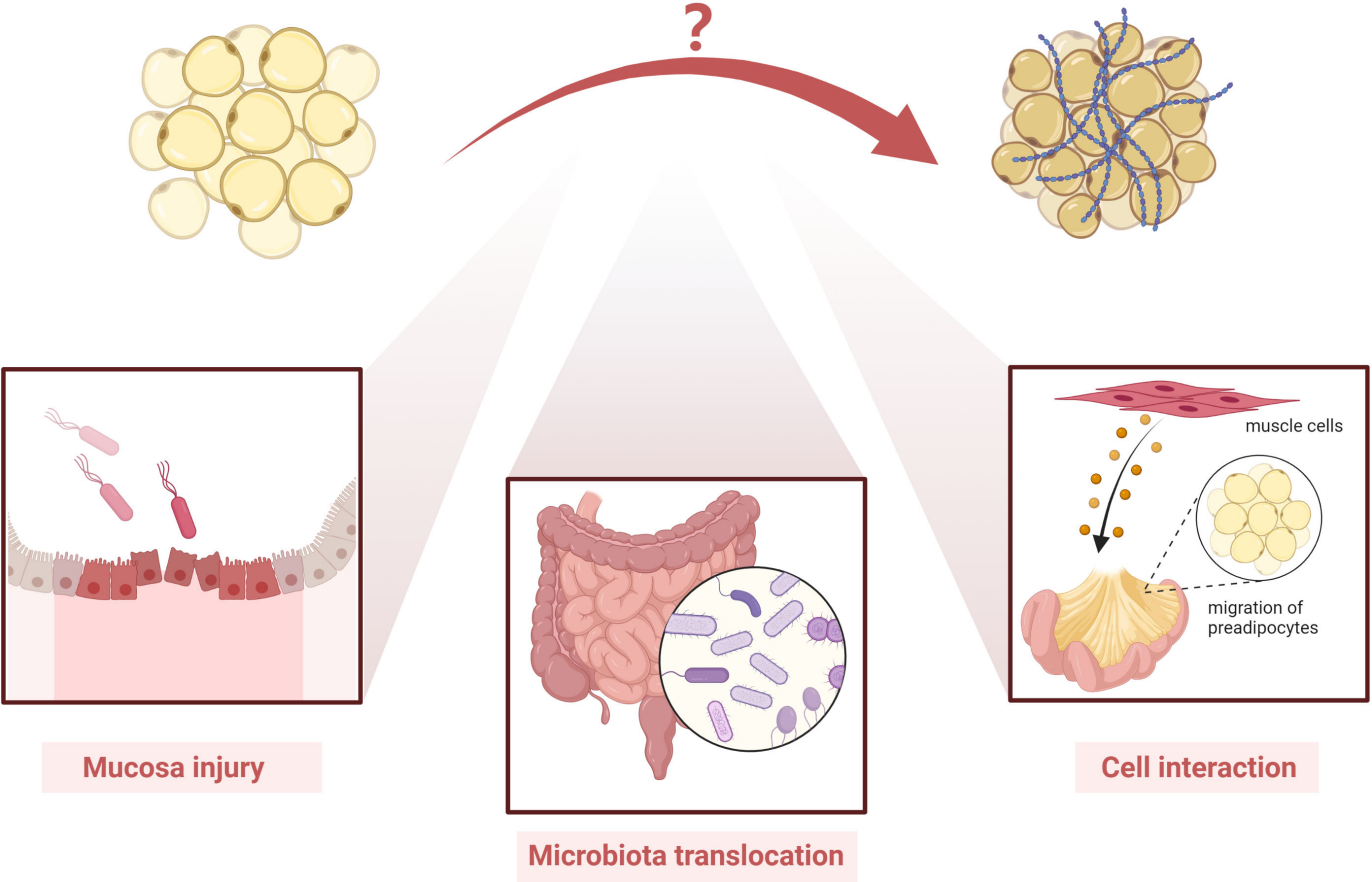
Possible players involved in the pathogenesis of creeping fat fibrosis. Although the pathogenesis of creeping fat fibrosis remains unclear, according to the current literature, mucosal injury, microbiota translocation, cell interaction, and activated intestinal muscle cells are important players in creeping fat formation. However, other mechanisms need to be investigated in future studies.

In another study by our team, we used novel intestinal tissue and cell interaction systems to illustrate that muscle cells in CD patients could produce an ECM scaffold that triggered preadipocyte migration out of the mesenteric adipose tissue (6). This finding highlights that cell–cell interaction and activated intestinal muscle cells are important players in creeping fat formation (**Figure 3**) (6).

Recently, Zuo et al. also observed aberrant ECM remodeling in the mesenteric adipose tissue of CD. This area served as a reservoir for inflammatory cells and factors. Moreover, TLR4-mediated macrophages were shown to play a role in mesenteric ECM remodeling and thus affecting the adipocyte function. They further validated the function of macrophages and TLR-4 using *in vivo* and *in vitro* experiments (61).

All in all, various cells, including fibroblasts, smooth muscle cells, preadipocyte, and macrophages, are involved in the fat fibrosis of CD. The mechanisms need further study.

## 6 Discussion

In summary, adipose tissue fibrosis in creeping fat is a complex phenomenon involving various cytokines and cellular interactions. The findings from other diseases will help us functionally investigate the latent mechanisms of creeping fat fibrosis. However, there is still a lot to unravel to completely understand this process. As a characteristic manifestation in CD, creeping fat occurs in tandem with abnormal mural and mucosal changes. The functional changes in fibrotic mesenteric fat and how these functional changes affect CD disease activity in the adjacent intestinal segments still need to be addressed. It is of great importance in future studies to investigate the specific genes, cells, and putative mechanism that play a role in the fibrotic process of creeping fat. We believe that understanding the mechanism behind adipose tissue fibrosis in creeping fat will help develop novel targets for the diagnosis and treatment of CD.

## Author contributions

RM and ZC conceived the idea. SX and JT performed the literature search and drafted the manuscript. SX and YW drafted the figures. All authors contributed to the revision of the manuscript. RM supervised the study. All authors contributed to the article and approved the submitted version.

## Funding

This work was supported by the National Natural Science Foundation of China (NSFC grant Nos. 81970483, 82170537 and 82222010 to RM).

## Acknowledgments

All figures are created with BioRender.com.

## Conflict of interest

The authors declare that the research was conducted in the absence of any commercial or financial relationships that could be construed as a potential conflict of interest.

## Publisher’s note

All claims expressed in this article are solely those of the authors and do not necessarily represent those of their affiliated organizations, or those of the publisher, the editors and the reviewers. Any product that may be evaluated in this article, or claim that may be made by its manufacturer, is not guaranteed or endorsed by the publisher.

## Abbreviations

AF, atrial fibrillation; CD, Crohn’s disease; ECM, extracellular matrix; HIF1α, hypoxia-inducible factor 1-alpha; IL, interleukin; ILC1s, group 1 innate lymphoid cells; MMP, matrix metalloproteinase; PDGF, platelet-derived growth factor; PPARγ, peroxisome proliferator-activated receptor-gamma; TGF-β1, transforming growth factor-beta 1; TIMP, tissue inhibitors of MMP.
